# Using Hospital Antibiogram Data To Assess Regional Pneumococcal Resistance to Antibiotics

**DOI:** 10.3201/eid0902.020123

**Published:** 2003-02

**Authors:** Cheryl R. Stein, David J. Weber, Meera Kelley

**Affiliations:** * The University of North Carolina at Chapel Hill, Chapel Hill, North Carolina

**Keywords:** drug resistance, microbial, population surveillance, *Streptococcus pneumoniae*, penicillin resistance, macrolides, research

## Abstract

Antimicrobial resistance to penicillin and macrolides in *Streptococcus pneumoniae* has increased in the United States over the past decade. Considerable geographic variation in susceptibility necessitates regional resistance tracking. Traditional active surveillance is labor intensive and costly. We collected antibiogram reports from North Carolina hospitals and assessed pneumococcal susceptibility to multiple agents from 1996 through 2000. Susceptibility in North Carolina was consistently lower than the national average. Aggregating antibiogram data is a feasible and timely method of monitoring regional susceptibility patterns and may also prove beneficial in measuring the effects of interventions to decrease antimicrobial resistance.

*Streptococcus pneumoniae* is a leading cause of community-acquired illness, resulting in an estimated 3,000 cases of meningitis, 50,000 cases of bacteremia, 500,000 cases of pneumonia, and 7 million cases of otitis media each year in the United States (*1*). Even with appropriate antimicrobial therapy, case-fatality rates for high-risk patients can be as high as 40% for bacteremia and 55% for meningitis (*1*). Surveillance systems have shown decreasing antimicrobial susceptibility among pneumococci (*2*–*13*). A comparison of susceptibility among combined respiratory and invasive isolates from respiratory seasons 1994–1995 and 1999–2000 showed a decrease in both penicillin susceptibility (from 76% to 66%) and erythromycin susceptibility (from 90% to 74%) (*8*). An examination of only invasive isolates from 1997 and 2000 also showed a decline in isolates’ susceptibility to penicillin (from 75% to 73%) and to erythromycin (from 85% to 78%) (*2*,*5*).

Many of the current surveillance systems monitoring emerging drug resistance detect susceptibility patterns over large areas, such as an entire country (*2*,*6*,*9*,*13*). Results may not be generalizable to all locations within the study area (*1*). For example, although the overall proportion of penicillin-nonsusceptible pneumococci within a seven-region, population-based, active surveillance program was 25%, the proportion ranged from 15% in Maryland to 38% in Tennessee (*14*). Data are typically gathered from a limited number of medical establishments within a specified region; national surveillance systems may not collect data from every state. Monitoring trends in pneumococcal susceptibility over smaller geographic areas is necessary to aid clinicians in choosing the best drug treatment for empiric therapy (*1*). This local information can also help evaluate efforts to decrease resistance rates through judicious antibiotic use.

Chin et al. compared antimicrobial susceptibility results from active and antibiogram surveillance of pneumococcal isolates at 12 hospitals in the Portland, Oregon, area in 1996 (*15*). Active surveillance was defined as collecting isolates and patient data from participating hospitals and performing susceptibility testing at a centralized laboratory (*15*,*16*). Antibiogram surveillance is quite different. Clinical laboratories assess the antimicrobial susceptibilities of bacterial isolates and summarize all susceptibility results for a specified period on an antibiogram report. Antibiograms conform to the susceptibility testing practices of individual laboratories, include information on both sterile and nonsterile isolates, may include duplicate isolates from a single patient, and lack an epidemiologic characterization of the patient or isolate. The data contained on laboratory-specific antibiograms, however, can be compiled to assess regional susceptibility, monitor trends over time, and assess effects of interventions designed to reduce antibiotic resistance through judicious antibiotic use.

Chin et al. found no statistically significant difference in results obtained by the two methods for any of the four drugs tested at the 12 Portland area hospitals, except for a single drug at a single hospital where erythromycin susceptibility was reported at 97% by active surveillance and 84% from the antibiogram (chi square p=0.01) (*15*). The cost difference between the active and antibiogram surveillance systems was substantial: $52,000 for active surveillance and $700 for antibiogram surveillance. The authors concluded that although antibiogram surveillance produced less information than active surveillance, “antibiograms provided accurate, community-specific drug-resistant *S. pneumoniae* data.”

We examined the practicability of collecting hospital antibiogram data in North Carolina, a state with a population of 8,049,313 people and a land area of 48,711 square miles divided into 100 counties (*17*). We also assessed pneumococcal susceptibility to multiple antimicrobial agents using the aggregated antibiogram data.

## Methods

This study was conducted in North Carolina from April to September 2001. A study packet was mailed in April 2001 to the directors of clinical microbiology laboratories at all 114 North Carolina hospitals identified by the Centers for Medicare and Medicaid Services (CMS). Hospitals subsequently identifying themselves as specialty hospitals (e.g., psychiatric, drug treatment) were excluded from all analyses. The packet included a letter describing the project, a questionnaire on hospital characteristics and laboratory testing methods, a request for submission of *S. pneumoniae* antibiogram data for each year from 1996 to 2000, and a prepaid, preaddressed, return express mail envelope. The nature of the information provided on hospital antibiograms does not necessarily allow for identification of duplicate specimens from the same patient, differentiation of susceptibility results by source of specimen, or determination of conformance to National Committee for Clinical Laboratory Standards (NCCLS) guidelines for susceptibility cut points.

From the antibiograms, the numbers of pneumococcal isolates tested and numbers of isolates testing susceptible were added across all hospitals for each antimicrobial agent for each year of data to create statewide summary totals. Data for drugs that predictably elicit the same susceptibility result were combined: penicillin/oxacillin, cefotaxime/ceftriaxone, and levofloxacin/ofloxacin. Antibiogram data from each hospital for each year of data were assessed for inclusion. Data from antibiograms reporting 1) testing results cumulative over >1 year, 2) percentages of susceptible isolates without providing the total number of isolates tested, 3) more than one susceptibility value for the same drug for the same period, or 4) results by nursing unit or named patient were all excluded for the year and drug in question. We also excluded data for drugs other than penicillin if more isolates were tested for penicillin susceptibility than for the other drugs, and the antibiogram did not clearly indicate that the subgroup selected for additional testing was based on the source of the specimen (i.e., bloodstream). Testing only penicillin-nonsusceptible isolates for susceptibility to other drugs could yield misleading results because penicillin-resistant isolates are more likely to be resistant to other drugs as well (*8*).

The aggregated statewide summary totals were used to calculate yearly susceptibility proportions for nine different antibiotics for the entire state. Nonsusceptible isolates encompassed those identified as either intermediate- or high-level resistant. Susceptibility proportions for penicillin were also stratified by geographic region of the state. Hospitals were categorized into three regions (west, central, east) by the county the respondent listed on the questionnaire. The Committee on the Protection of the Rights of Human Subjects, University of North Carolina School of Medicine, granted Institutional Review Board approval.

North Carolina’s pneumococcal susceptibility pattern from 1997 through 2000 was compared to patterns shown by national surveillance systems tracking *S. pneumoniae* susceptibility. Data from published reports were included if they covered a period of no more than 12 months and identified the source of isolates as respiratory, invasive, or both. If the surveillance period overlapped two calendar years while covering one respiratory season, the data were classified by the latter year. For instance, isolates collected from October 1999 through April 2000 were labeled as year 2000 data.

We used the Cochran-Armitage trend test, which tests for trends in binomial proportions across levels of an ordinal covariate, to evaluate temporal patterns in the data. A two-sided p-value <0.05 was considered statistically significant. Trend tests were performed by using SAS version 8.1 (SAS Institute, Inc., Cary, NC). Exact binomial 95% confidence intervals were calculated for proportions by using Stata version 7.0 (Stata Corporation, College Station, TX).

## Results

Overall, 60 of the 110 (55%) potentially eligible hospitals replied to the survey, although ultimately fewer hospitals were able to contribute pneumococcal susceptibility data. Thirty of the 114 CMS-identified hospitals responded to the initial request for information within the first month. After follow-up telephone calls to the remaining 84 hospitals, 30 additional hospitals responded. Four hospitals were excluded: one listed under two different names, one psychiatric hospital, one orthopedic hospital, and one alcohol treatment center. North Carolina hospitals not enumerated on the CMS list, and hence not invited to participate in this study, included five military, four Veterans Affairs, and two prison hospitals, as well as several long-term care and rehabilitation centers. Additionally, small hospitals that were part of a health-care system dominated by one large hospital were frequently omitted from the CMS list. These noninvited hospitals were primarily rural, community facilities.

The proportion of responding hospitals was similar across the three regions of the state: 17/30 (57%) hospitals in the west, 27/51 (53%) in the central region, and 16/29 (55%) in the east. The average number of beds was 257 (range 40 to >1,000). The central region contained most of the state’s large, academic hospital centers as well as most of its urban counties. No discernable difference was evident between the 50 potentially eligible hospitals that did not participate and the 60 that did, except that all major academic centers participated.

The primary source of pneumococcal isolates was specimens from hospitalized patients in 74% of hospitals and outpatients in 12%. The remaining specimens came from emergency departments, nursing homes, and physicians’ offices. Among the hospitals describing susceptibility testing methods, 51% used E-test, 47% oxacillin screening, 36% MIC broth dilution, and 20% disk diffusion. Many hospitals performed more than one type of susceptibility testing. Although 11 hospitals reported differentiating sterile from nonsterile isolates, only 7 hospitals provided this stratification on their antibiograms.

Eleven of the 60 (18%) hospitals responding to the study request did not provide antibiogram data. Of these hospitals, nine did not perform on-site susceptibility testing, and two gave no explanation for not submitting antibiograms. One additional hospital reporting off-site testing made antibiogram information available through a reference laboratory. Although 49 hospitals contributed antibiogram data for at least one drug for at least 1 year, not all of the data from these antibiograms qualified for inclusion in the analyses. Susceptibility data were excluded for at least one class of drugs for >12 months from 1996 to 2000 for the following reasons: 1) seven antibiograms provided data for a period of more than 12 months, 2) four antibiograms reported susceptibility rates without numbers of isolates tested,3) one antibiogram listed more than one susceptibility result for the same drug for the same period, 4) two antibiograms provided results by nursing unit or named patient, and 5) 12 antibiograms reported more isolates tested for penicillin susceptibility than for susceptibility to other drugs without explaining the drop-off in isolate number, therefore, requiring exclusion of the nonpenicillin data. After accounting for the aforementioned exclusions, the number of antibiograms with usable data for any single drug for a given 1-year period ranged from 1 (levofloxacin, 1996 and 1997) to 42 (penicillin, 2000).

Although most hospitals submitted susceptibility testing results for 2000, fewer did so for earlier years. The numbers of hospitals providing data on penicillin susceptibility were 18 hospitals (1,854 isolates) for 1996, 24 hospitals (2,406 isolates) for 1997, 33 hospitals (2,827 isolates) for 1998, 36 hospitals (3,562 isolates) for 1999, and 42 hospitals (3,497 isolates) for 2000 (Table 1). The numbers of hospitals submitting data on macrolide susceptibility also increased over the years: four hospitals (488 isolates) for 1996, 11 hospitals (786 isolates) for 1997, 17 hospitals (1,095 isolates) for 1998, 20 hospitals (1,397 isolates) for 1999, and 27 hospitals (1,762 isolates) for 2000 (Table 1).

**Table 1:** *Streptococcus pneumoniae* susceptibility to nine antimicrobial agents, North Carolina, 1996–2000

Year	Percent of all isolates susceptible to^a^								
	Penicillin	Erythromycin	Cefotaxime	Levofloxacin	Tmp-smx^b^	Tetracycline	Clindamycin	Vancomycin	Chloram-phenicol
1996	65 (18; 1,854)	78 (4; 488)	85(9; 985)	100 (1; 205)	64 (5; 626)	96 (2; 254)	90(4; 492)	100 (7; 580)	93 (2; 381)
1997	63 (24; 2,406)	69 (11; 786)	80 (13; 1,272)	100 (1; 283)	57 (11; 786)	79 (4; 66)	76 (7; 655)	100 (11; 903)	95 (3; 117)
1998	56 (33; 2,827)	64 (17; 1,095)	83 (20; 1,970)	92 (4; 237)	51 (16; 975)	83 (10; 402)	88 (11; 606)	100 (19; 1202)	89 (10; 520)
1999	54 (36; 3,562)	61 (20; 1,397)	80 (22; 2,062)	94 (5; 525)	51 (16; 1,068)	84 (10; 406)	85 (13; 1,017)	100 (21; 1308)	92 (9; 540)
2000	52 (42; 3,497)	61 (27; 1,762)	77 (27; 2,296)	98 (12; 822)	50 (20; 1,292)	81 (14; 717)	88 (18; 1,238)	100 (26; 1,648)	94 (12; 730)

^a^Values in parentheses are number of hospitals contributing data and total number of isolates reported, respectively

^b^Tmp-smx, trimethoprim-sulfamethoxazole

**Figure 1:**
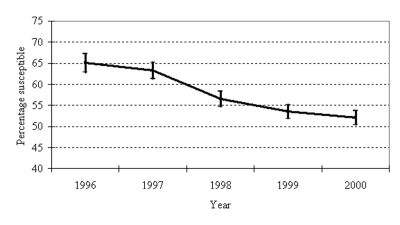
*Streptococcus pneumoniae* penicillin susceptibility, North Carolina, 1996–2000. Error bars represent 95% confidence intervals.

**Figure 2:**
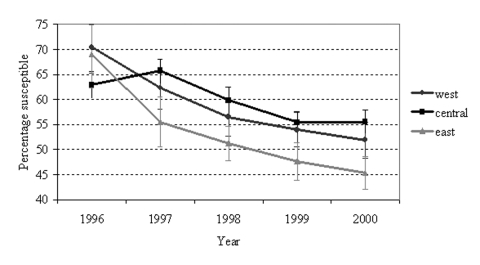
*Streptococcus pneumoniae* penicillin susceptibility by geographic region, North Carolina, 1996–2000. Error bars represent 95% confidence intervals.

From 1996 to 2000, the proportion of *S. pneumoniae* isolates testing susceptible to penicillin decreased (p<0.001) (Figure 1). Although 65% of isolates were reported as susceptible to penicillin in 1996, only 52% were susceptible in 2000 (p<0.001). This pattern of decreasing susceptibility was also evident when stratifying by region of the state (p<0.001 for each region) (Figure 2). From 1997 on, no statistically significant difference in susceptibility was found between either the west and eastern regions or the west and central regions. However, penicillin susceptibility was significantly lower (10%) in the east than in the central region during this time period.

A subanalysis of the 15 hospitals for which usable information on penicillin susceptibility was available for each year produced a comparable trend. Although with this reduced amount of data susceptibility was higher in 1996 (69%) than in the full analysis (65%) (chi square p=0.03), the trend over time remained consistent (p<0.001). By 2000, 54% of the isolates reported at these 15 hospitals were susceptible to penicillin, compared to 52% in the entire study group (chi-square p=0.13).

**Figure 3:**
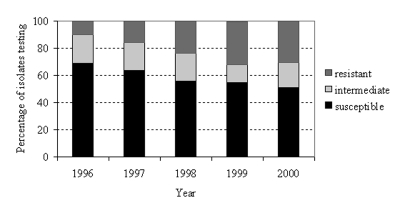
*Streptococcus pneumoniae* penicillin susceptibility among isolates differentiating nonsusceptibility levels, North Carolina, 1996–2000.

Among penicillin-nonsusceptible isolates, the proportions intermediate and resistant were available from 5 hospitals (419 isolates) in 1996, 6 hospitals (592 isolates) in 1997, 11 hospitals (849 isolates) in 1998, 12 hospitals (1,184 isolates) in 1999, and 11 hospitals (1,055 isolates) in 2000. These represented between one-quarter and one-third of all isolates tested for penicillin susceptibility, depending on the year. During the 5-year period, the proportion of susceptible isolates appeared to decrease, the proportion of resistant isolates increased, and the proportion of intermediate isolates showed little change (Figure 3).

Macrolide susceptibility decreased from 78% in 1996 to 61% in 2000 (p<0.001). From 1996 to 2000, the proportion of *S. pneumoniae* isolates susceptible to cefotaxime decreased 8%. Although third-generation cephalosporins did not show a consistent decrease in susceptibility each year, the decline during the 5-year period was still significant (p<0.001). Susceptibility to quinolones and vancomycin remained high, despite the fact that two hospitals, one for two different years, reported a total of five isolates as vancomycin-resistant. The low level of levofloxacin susceptibility in 1998 (92%) was based on only 237 isolates. Larger numbers of isolates available in subsequent years did not support a pattern of greatly reduced susceptibility.

## Discussion

Aggregating hospital antibiogram data from the state of North Carolina appears to be a feasible, practical method for monitoring trends in pneumococcal susceptibility. Large numbers of isolates are available for annual comparisons with consistent reporting on penicillin, albeit less consistent reporting on other drugs. Susceptibility to clinically important antibiotics was shown to decrease significantly.

The observed progression of both penicillin and macrolide resistance is of particular concern. The increase in penicillin resistance appears to correlate with an increase in high-level rather than intermediate-level resistance and high-level penicillin resistance has been associated with worse outcomes for pneumococcal infections (*18*). The increased macrolide resistance is most likely mediated by a low-level efflux pump since clindamycin susceptibility remained stable over the study period (*19*). Erythromycin susceptibility generally predicts that of azithromycin and clarithromycin (*20*). Increased macrolide resistance is disturbing since erythromycin, azithromycin, and clarithromycin are some of the most commonly prescribed antibiotics for outpatient treatment of community-acquired pneumonia and low-level macrolide resistance has been associated with clinical failure (*21*–*23*).

Pneumococcal resistance rates tend to increase moving along the spectrum of isolates obtained from bloodstream to lower respiratory tract to upper respiratory tract (*8*). This fact potentially confounds point comparisons of resistance rates since a marked increase in resistance can result from testing a preponderance of upper respiratory isolates, rather than reflecting a true increase in the burden of resistant pneumococci. We were unable to assess the extent to which the source of the specimen may have produced spurious results since only seven hospitals identified the specimen source on their antibiograms. If the relative distribution of isolates remained the same, however, the trend would not be altered.

Although Chin et al. showed that antibiogram surveillance and active surveillance yield comparable results, national data may not be directly comparable to our findings (*15*). The national data used for comparison to this study result from active surveillance use different reporting periods, and in some cases focus solely on invasive isolates that have consistently higher susceptibility than respiratory isolates (*8*). Pneumococcal susceptibility in North Carolina is nonetheless lower than reported national averages and appears to be decreasing more quickly (Table 2). This finding is consistent with comparatively low antibiotic susceptibilities previously described for the Southeast. Active surveillance for year 2000 susceptibility in the southeastern United States ranged from 56% to 57% for penicillin and from 61% to 67% for erythromycin (*8*,*12*,*13*).

**Table 2:** Comparison of surveillance systems tracking *Streptococcus pneumoniae* susceptibility to select antimicrobial agents, United States, 1997–2000^a^

		Percentage of all isolates susceptible to								
Year	Study^c^	Penicillin	Erythro-mycin^d^	Cefotaxime	Levo-floxacin^e^	Tmp-smx^d^	Tetracyline	Clinda-mycin	Vanco-mycin	Chloram-phenicol
1997	ABC (2–5) TRUST (9–12)	75 67	85 81	87 87	96 97	71 ––	–– ––	–– ––	100 ––	–– ––
	NC	63	69	80	100	57	79	76	100	95
1998	ABC Doern (7–8) TRUST	76 70 65	85 81 77	86 –– 88	100 98 100	71 69 68	–– 87 ––	–– 94 ––	100 –– 100	–– 93 ––
	NC	56	64	83	92	51	83	88	100	89
1999	ABC TRUST	73 67	80 77	83 85	100 99	68 66	–– ––	–– ––	100 100	–– ––
	NC	54	61	80	94	51	84	85	100	92
2000	ABC Doern RESP (13) TRUST	73 66 84 66	78 74 66 73	82 –– 95 83	100 99 100 99	68 64 70 65	–– 83 80 ––	–– 81 89 93	100 –– 100 100	–– 92 –– ––
	NC	52	61	77	99	50	81	88	100	94

^a^Tmp-smx, trimethoprim-sulfamethoxazole; ABC, Active Bacterial Core Surveillance; TRUST, Tracking Resistance in the United States Today; RESP, Respiratory Surveillance Program.

^b^If the surveillance overlapped two calendar years while covering one respiratory season, data were classified by the latter year. For example, isolates collected from October 1999 to April 2000 were labeled as year 2000 data.

^c^Data for only invasive isolates (ABC); respiratory and invasive isolates (Doern, TRUST, NC); only respiratory isolates (RESP)

^d^1997–1999 TRUST data are for clarithromycin.

^e^1997 ABC data are for ofloxacin; all Doern data are for ciprofloxacin.

Antibiogram surveillance is cost efficient. Expenses for collecting and analyzing five years worth of data from an entire state were limited to mailings and paper materials (<$1,000) and the support of one graduate student. Chin et al. spent $52,000 for 1 year of active surveillance in 12 hospitals. The antibiogram surveillance had several potential limitations, however. First, a 55% response rate may be adequate for certain surveys, but full participation from all N.C. microbiology laboratories would be the best way to ensure that surveillance data accurately reflect susceptibility patterns. Furthermore, all participating hospitals did not submit an antibiogram for each year nor did all data on each antibiogram meet inclusion criteria. Yet our data included many more hospitals in this study locale than any previously published surveillance system. Second, many hospitals were unable to access data from past years for a variety of reasons, including changes in testing and computer systems. Collecting antibiogram reports on a yearly basis should allow more hospitals to more easily contribute their data. Third, specimens are increasingly sent to referral hospitals or reference laboratories. For instance, 9 of the 60 participating hospitals did not have antibiogram data, and we were able to get results from a reference laboratory for only a single hospital. The lack of availability of antibiograms at hospitals that use reference laboratories is disconcerting since information needed to guide local antibiotic decisions is not accessible. Lastly, testing and reporting procedures were inconsistent, such as drugs tested, identification of specimen source, breakdown of intermediate and high-level resistance, and period covered by the antibiogram. We hope that providing N.C. microbiology laboratories with these study findings will encourage participation in continued surveillance activities as well as more uniform testing and reporting procedures.

The submitted data appeared to be consistent despite the fact that the population under study changed from year to year as more data became available. The results of the penicillin sub-analysis, consisting solely of those hospitals providing usable information for each year from 1996 to 2000, yielded results comparable to those found in the overall study. Additionally, the observed susceptibility results generally support known resistance patterns, such as the correlation of penicillin and ceftriaxone susceptibility, lower levels of macrolide than clindamycin susceptibility, and near universal vancomycin susceptibility (*24*).

Combining hospital antibiogram data appears to be an effective method of tracking antimicrobial susceptibility among *Streptococcus pneumoniae*, both in North Carolina and within regions of the state. To further develop this antibiogram surveillance system, we are partnering with the North Carolina State Laboratory of Public Health to establish an electronic network of microbiology laboratories to enhance interlaboratory communication. We hope to share practices, encourage testing consistent with current NCCLS guidelines, and support standardized, efficient, annual, electronic submission of antibiograms. We also hope that knowledge of N.C. resistance patterns will both guide treatment decisions and motivate judicious antibiotic prescribing behavior.

Judicious use of antibiotics is essential for their continued effectiveness. Not only have regional trends in antibiotic resistance been linked to antibiotic use (*25*–*29*), but decreasing antibiotic use has resulted in declining levels of resistance (*30*). After an aggressive campaign to educate its population on the need for shrewd use of antibiotics, the rate of penicillin-resistant pneumococci in Iceland declined from nearly 20% in 1993 to 16.9% in 1994 (*31*,*32*). Regional surveillance can identify areas most in need of interventions aimed at decreasing resistance and can monitor the progress of these interventions. Aggregating antibiogram data appears to be an easy, inexpensive, effective way of accomplishing these goals.
